# Data sets on delineation of groundwater potential zones identified by geospatial tool in Gudur area, Nellore district, Andhra Pradesh, India

**DOI:** 10.1016/j.dib.2018.09.054

**Published:** 2018-09-26

**Authors:** Veeraswamy Golla, Balaji Etikala, Ala Veeranjaneyulu, M. Subbarao, A. Surekha, K. Narasimhlu

**Affiliations:** a Dept. of Geology, Sri Venkateswara University, Tirupathi, Andhra Pradesh, India; b Dept. of Geology, Acharya Nagarjuna University, Guntur, Andhra Pradesh, India; c Dept. of Geography, Sri Venkateswara University, Tirupathi, Andhra Pradesh, India; d Dept. of Civil Engineering, AITS, Tirupathi, Andhra Pradesh, India

**Keywords:** Geology, Geomorphology, Lineament, Drainage, Slope map and Hydro geomorphology map

## Abstract

The data deals with the preparation of the groundwater potential zone map of gudur area, with the help of data like geology and geomorphology, structure/lineament, slope and drainage and the thematic layer were prepared through the Survey of India toposheet Nos. N/12,N/15,N/16 and IRS-P6 LISS-III(RESOURCESAT-2) satellite data. The groundwater potential zones were obtained and classified into four categories, viz., very poor, poor, good, and very good zones. The data explains lateritic plain moderate basement with poor potential zones whereas secondarily occupies alluvial plain contains the good prospecting zone.

**Specification table**

**Table:** 

Subject area	Hydro Geology and Remote sensing and GIS
Specific subject area	Remote Sensing and GIS
Type of data	Table, Geospatial data and SRTM DEM, Toposheet
How data was acquired	LISS-III Satellite Imageries from Www.BHUVAN.NRSA Website, toposheets (GSI) and field surveys
Data format	Raw, analyzed
Experimental factors	Toposheets and Satellite imageries are georeferenced and digitized by using Arc GIS 10.1 & ERADAS imagine software
Experimental factors	Ground water potential zones
Data source	long 79°42′ 30′′E–79°54′30′′E and lat°13′0′′N14°16′30′′ N.
Data accessibility	Data is within this articles

**Value of the data**

• This data can serve as the base line for studies of that area.
• The data is helpful for the geologist, hydro geologist and as well as irrigation water supply Engineers forecasting the ground water availability.
• The data can be used to provide details about the geology, land use/land cover slope drainage geomorphology and hydrogeomorphology of the study area.

## 1 Data

Data sets like geology, geomorphology, land use land cover were derived from IRS-P6 LISS-III (RESOURCESAT-2) satellite data and drainage, lineaments and slope data from toposheet (GSI), SRTM DEM data, respectively. *Specification Table* presents the source of dataset downloads and Table 1.1 presents the relationship between thematic layers.

**Table 1.1:** The occurrence of ground water based on the geomorphic units and its characteristics in Gudur area.

**Geomorphic unit**	**Characteristics**	**Hydro geology**	**Ground water potential**
Pedi plain Moderate Weathered (PPM)	It is a weathered, fractured rock	Biotite granite gneisses weathered	Good
Linear ridge (LR)	Intrusion of igneous body cutting across existing strata	Quartzite	Negligible
Lateritic plain deep basement (LPD)	It is a weathered rock, fissured rock	Migmatised granetiferous quartz mica schist with amphibolite bands	poor
Lateritic plain moderate Basement (LPM)	It is a weathered rock, fissured rock	Migmatised granetiferous quartz mica schist with amphibolite bands	Poor to moderate
			
Alluvial plain moderate (APM)	Flat surface adjacent to stream/river, composed by Clay, Silt and Sand these are paleo channels, channel bars	Alluvium, sand, silt	Very good
		Clay dominated	
Inselberg (I)	It is a massive isolated hill	Quartz sericite schist. chlorite schist, hornblende schist	Run off zone
Pediment (PD),	It is a flat surface of Pedi plain of granite gneiss and schist with 20–60 m. thick weathered material and covered with soils. It covered commonly the topographically low areas near stream courses and associated with fractured/lineaments.	Calc silicate rock, quartzite	Poor
Denudational hill (DH)	Broad uplands of considerable elevation, steeply sloping on all	Quartzite	negligible
Valley fill shallow weathered (VFS)	Valley fill shallow weathered material sand, silt, clay	Unconsolidated Sediment(sand, silt, clay)	Very good

### 1.1 Study area

**Fig. 1.0:**
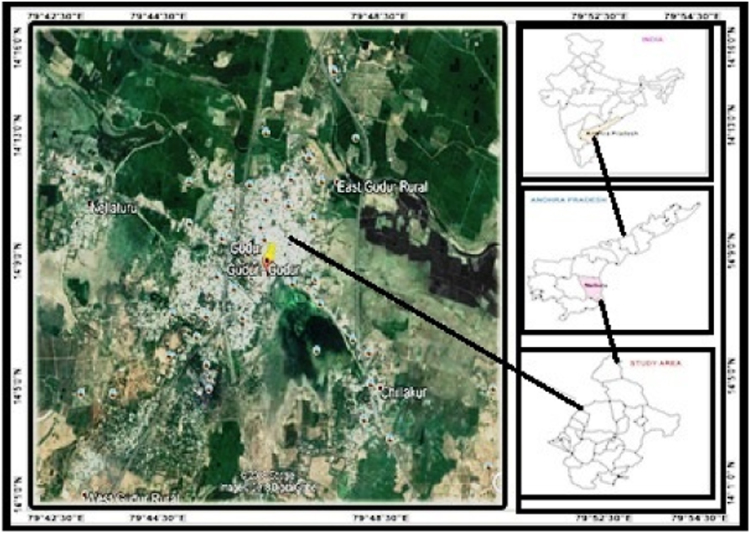
Location map of the study area.

Gudur area is the one of the Mandal in Nellore district of Andhra Pradesh. The area is situated in the coast on the Bay of Bengal, it is delimited by Balayapalli, Sydapuram, Manubole, Ozili, and Chillakuru mandal and possess world richest mica (muscovite) deposits in Nellore schist belt, and also quartz, feldspars deposits and covers an area of 247.29 km^2^. It is located between longitudes 79°42′30′′E–79°54′30′′E and latitudes 14°13′00′′N–14°16′30′′N. The annual average rainfall contribution by the northeast monsoon, increases from west to east about 900 to 1300 mm (Fig. 1.0).

## 2 Experimental design, materials and methods

Thematic maps such as drainage, geology, geomorphology, land use/land cover, structural lineament and hydrogeomorphology maps have been generate [1], [2], [3], [4], [5]. A drainage map has been generated from the SRTM (shuttle radar terrain model) digital elevation model data from USGS EARTH EXPLORER, Geology map has been generated from the District Geology and Minerals resource map of Nellore district (2001) and Soil map has been generated from the soil maps of Gudur area, and Nellore districts published by the Andhra Pradesh state Agricultural Department (2002). The preparation of geomorphology, land use/land cover maps were prepared from -IRS-P6 LISS-III (RESOURCESAT-2) data. Lineaments were extracted from toposheet taking into consideration of drainage pattern. The output data was draped on to the satellite image for its extension and best fit [6]. In addition to that extensively field checks were done in the study area.

### 2.1 Geology

**Fig. 2.0:**
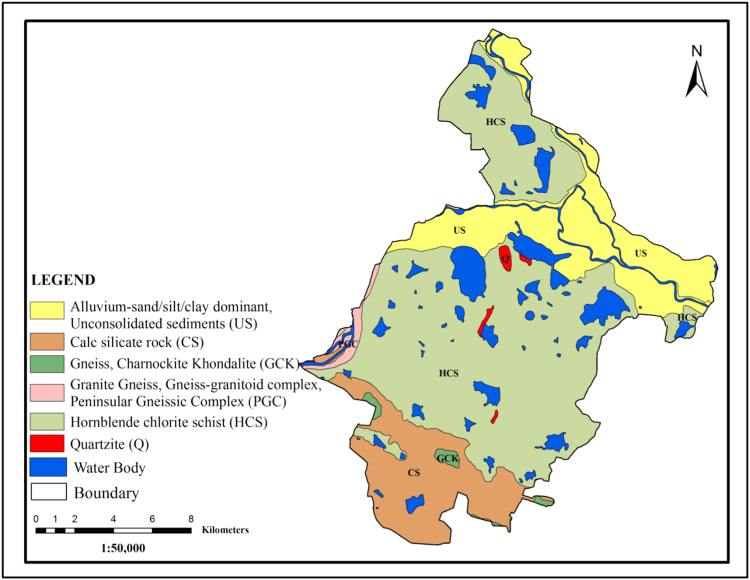
Geology map of the study area.

The Nellore schist belt extends parallel to the Eastern border of the cuddapah basin as an N-S trending actuate belt over a length of 180 km between Podili (Prakasam district) in the north and Naidupeta in the south, with width ranging between 5 km and 501 km. Here, geology was comprised the Hornblende chlorite schist, quartzite׳s, Gneisses, Granite gneisses, gneisses granitoid complex, peninsular gneissic complex, Calc-silicate rocks and unconsolidated alluvial sediments (Fig. 2.0).

### 2.2 Geomorphology

**Fig. 3.0:**
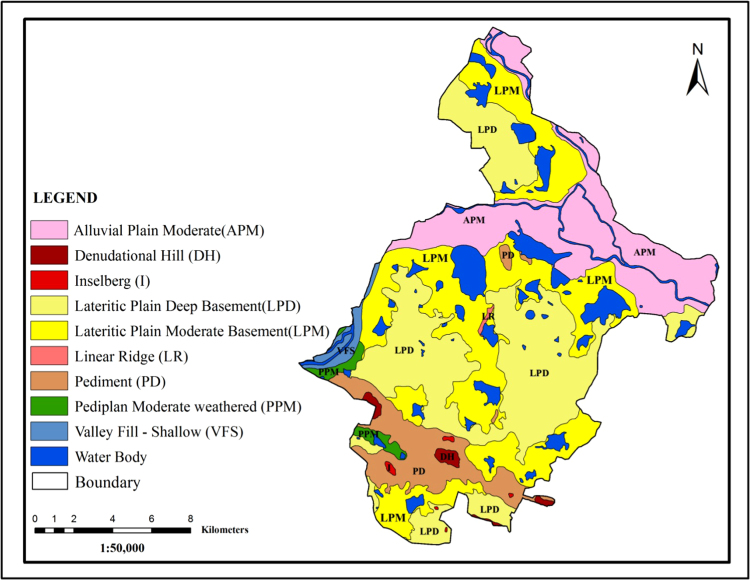
Geomorphology map of the study area.

The prominent geomorphic units have been identified in the area through interpretation of Satellite Imagery are Alluvial Plain Moderate (APM), Linear Ridge (LR), Pediment, inselberg, Lateritic plain deep basement (LPD), Valley fill shallow (VFS), Denudational1 hills (DH), Laterite plain moderate (LPM), Pedi plain Moderate weathered (PMW) and Water bodies. Among all geomorphic features, the valley fill contains high infiltration with a good groundwater potential zone (Fig. 3.0).

### 2.3 Structures/lineament

**Fig. 4.0:**
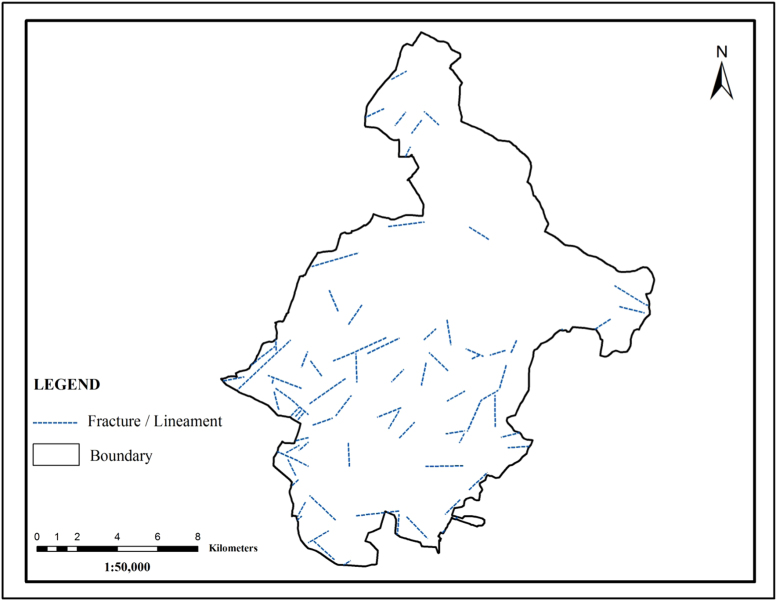
Lineament map of the study area.

Lineaments were structural control of the groundwater movement and played a vital role in the infiltration run off into the ground. In that present study area, 81 lineaments have been mapped through analysis of satellite data9 (Fig. 4.0). The analysis reveals that the majority of lineaments area oriented in Southwest to Northeast direction.

### 2.4 Slope

**Fig. 5.0:**
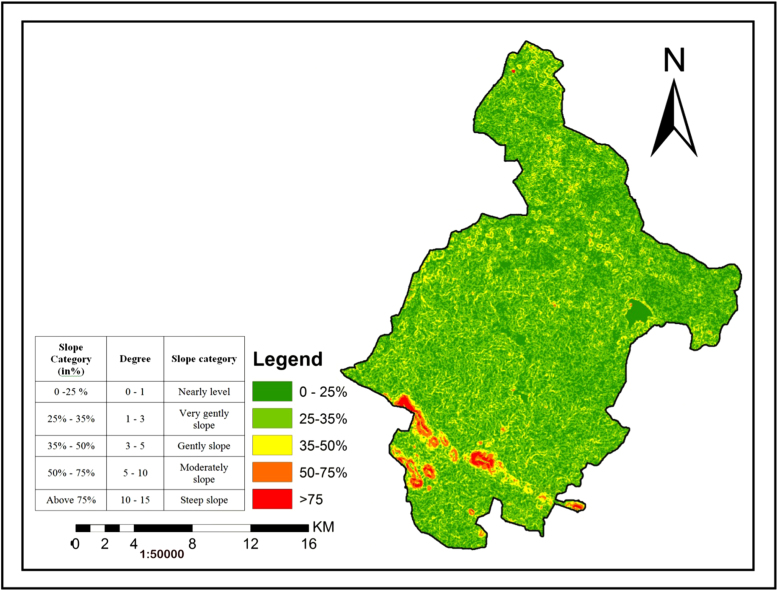
Slope map of the study area.

The slope map of the study area was prepared from SRTM Dem 30 m resolution to extract topographic information such as slope9 (Fig. 5.0). The analysis of slope map indicates that the study area covers five types of slope categories. The southern and south western parts of the study area has steep to gently slope and the remaining parts has nearly level to very gently slope [7].

### 2.5 Drainage

**Fig. 6.0:**
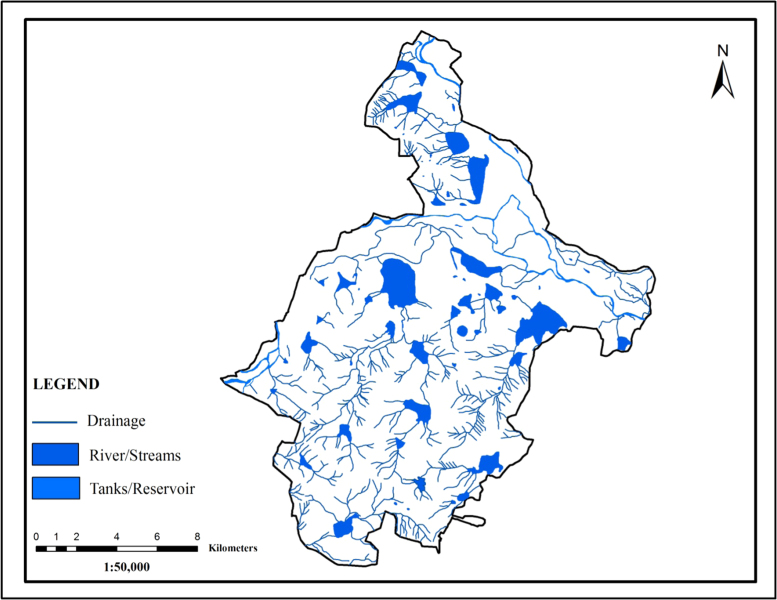
Drainage map of the study area.

The drainage map shows a Dendritic and Sub-dendritic drainage pattern is the most common pattern existing in the area which is characterized by irregular branching of tributary streams in many directions and at less than right angles. The patterns are observed to be developed in the granitic gneissic denudation hills and rolling pediplains of the study area, where structural control is negligible (Fig. 6.0). In the study area, four stream orders have been calculated, and 304 first order streams, 209 second order streams, 116 third order streams and 75 fourth order streams were derived.

### 2.6 Relationship of the data in groundwater potential zone map

**Fig. 7.0:**
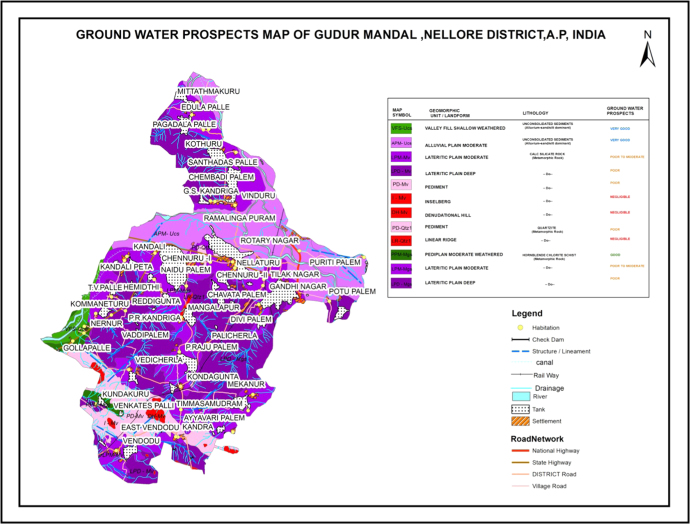
Groundwater potential map of the study area.

The hydro geomorphological map was prepared based on the specific tone, texture, size, shape and association characteristics of remotely sensed data. The purpose of elaborating a hydro geomorphological map intended to delineate the potential groundwater areas for the study region [8]. The occurrence of groundwater, based on the geomorphological units and its characteristics of the Gudur area is shown in the table 1.1 and shown Fig. 7.0.

## References

[1] Veeraswamy G., Nagaraju A., Balaji E., Sridhar Y. (2017). land use and land cover analysis using remote sensing and GIS: a case study in Gudur area, Nellore district, Andhra Pradesh. Int. J. Res..

[2] Nagaraju A., Balaji E., Padmanavadash (2016). Land use/land cover analysis based on various comprehensive geospatial data sets: a case study from Tirupati area, South India. Adv. Remote Sens..

[3] Magesh N.S., Chandrasekar N., Soundranayagam J.P. (2011). Morph metric evaluation of Papanasam and Manimuthar watersheds, parts of Western Ghats, Tirunelveli district, Tamil Nadu India: a GIS approach. Environ. Earth Sci..

[4] Krishnamurthy J., Srinivas G. (1995). Role of geological and geomorphological factors in groundwater exploration—a study through remote sensing techniques. Int. J. Remote Sens..

[5] Saraf A.K., Choudhary P.R. (1998). Integrated remote sensing and GIS for groundwater exploration and identification of artificial recharge sites. Int. J. Remote Sens..

[6] Narendra K., Nageswara Rao K., Swarna Latha P. (2013). Integrating remote sensing and gis for identification of groundwater prospective zones in the Narava Basin, Visakhapatnam Region, Andhra Pradesh. J. Geol. Soc. India.

[7] Shaban A., Khawlie M., Abdallah C. (2006). Use of remote sensing and GIS to determine recharge potential zone: the case of Occidental Lebanon. Hydrogeol. J..

[8] Pinto D., Shrestha S., Babel M.S., Ninsavat S. (2015). Delineation of groundwater potential zones in the Comoro watershed, Timor Leste using GIS, remote sensing, and analytic hierarchy process (AHP) technique. Appl. Water. Sci..

