# Supplementary material for: Data sets on delineation of groundwater potential zones identified by geospatial tool in Gudur area, Nellore district, Andhra Pradesh, India
Source: Data Brief. 2018 Sep 26;20:1984–91. doi: 10.1016/j.dib.2018.09.054 (PMC6171168; doi:10.1016/j.dib.2018.09.054)
Supplement: Supplementary file 1 — Supplementary material [file mmc1.docx]

Dear editor:

I don't have any conflict of as part my research work and publish of the data in your journal.

thank u sir

yours sincerely

GOLLA .VEERASWAMY
